# Levator Scapulae Stiffness Measurement Reliability in Individuals with and without Chronic Neck Pain by Experienced and Novel Examiners

**DOI:** 10.3390/s24010277

**Published:** 2024-01-03

**Authors:** Umut Varol, Juan Antonio Valera-Calero, Elena Sánchez-Jiménez, César Fernández-de-las-Peñas, Ricardo Ortega-Santiago, Mateusz D. Kobylarz, Marcos José Navarro-Santana

**Affiliations:** 1 Escuela Internacional de Doctorado, Universidad Rey Juan Carlos, 29222 Alcorcón, Spain; au.varol.2022@alumnos.urjc.es (U.V.); mateusz.kobylarz.fizjoterapia@gmail.com (M.D.K.); 2 Department of Radiology, Rehabilitation and Physiotherapy, Universidad Complutense de Madrid, 28040 Madrid, Spain; juavaler@ucm.es (J.A.V.-C.); marconav@ucm.es (M.J.N.-S.); 3 Grupo InPhysio, Instituto de Investigación Sanitaria del Hospital Clínico San Carlos (IdISSC), 28040 Madrid, Spain; 4 Faculty of Health, Universidad Católica de Ávila, 05005 Ávila, Spain; elena.sanchez@ucavila.es; 5 Department of Physical Therapy, Occupational Therapy, Rehabilitation and Physical Medicine, Universidad Rey Juan Carlos, 28922 Alcorcón, Spain; cesar.fernandez@urjc.es; 6 Cátedra Institucional en Docencia, Clínica e Investigación en Fisioterapia: Terapia Manual, Punción Seca y Ejercicio Terapéutico, Universidad Rey Juan Carlos, 28922 Alcorcón, Spain; 7 Akademia Terapii Manualnej i Igłoterapii Suchej (ATMIS), 34-400 Nowy Targ, Poland

**Keywords:** diagnostic accuracy study, levator scapulae, neck pain, shear wave elastography, ultrasound imaging

## Abstract

The levator scapulae muscle is a key structure in the etiopathology of neck and shoulder musculoskeletal pain. Although previous studies used shear-wave elastography (SWE) for characterizing this muscle elasticity, limited evidence assessed the inter-examiner reliability of this procedure. This study aimed to analyze the inter-examiner reliability for calculating Young’s modulus and shear wave speed in a cohort of participants with and without chronic neck pain. A diagnostic accuracy study was conducted, acquiring a set of SWE images at the C5 level in participants with and without neck pain (n = 34 and 33, respectively) by two examiners (one experienced and one novel). After blinding the participants’ identity, examiner involved, and side, the stiffness indicators were calculated by an independent rater in a randomized order. Intra-class correlation coefficients (ICC), standard error of measurement, minimal detectable changes, and coefficient of variation were calculated. Both cohorts had comparable sociodemographic characteristics (*p* > 0.05). No significant levator scapulae elasticity differences were found between genders, sides, or cohorts (all, *p* > 0.05). Inter-examiner reliability for calculating Young’s modulus and shear wave speed was moderate-to-good for assessing asymptomatic individuals (ICC = 0.714 and 0.779, respectively), while poor-to-moderate in patients with neck pain (ICC = 0.461 and 0.546, respectively). The results obtained in this study support the use of this procedure for assessing asymptomatic individuals. However, reliability estimates were unacceptable to support its use for assessing elasticity in patients with chronic neck pain. Future studies might consider that the shear wave speed is more sensitive to detect real changes in comparison with Young’s modulus.

## 1. Introduction

The levator scapulae muscle is anatomically located at the posterolateral and superficial aspect of the neck, originating from the posterior tubercles of C1–C4 transverse processes and reaching the superior and medial angle of the scapula (next to the minor rhomboid muscle insertion) [1,2]. However, multiple anatomical variations have been described [3,4]. For instance, imaging studies found accessory caudal attachments (to the serratus anterior and posterior superior and the first and second rib) in a considerable percentage of asymptomatic subjects [3], and anatomical studies found variations in the cranial attachments (to the mastoid process) [4]. The main functions of this muscle are to elevate and rotate inferiorly the scapula and the lateral flexion, ipsilateral rotation, and extension of the neck [5,6].

This muscle is highly interesting from a clinical point of view as consistent evidence supports its implication in the etiopathology of neck and shoulder musculoskeletal pain [7,8,9]. Previous studies reported functional findings in patients with rotator cuff tears and neck pain (i.e., limited scapular upward rotation at 90° of arm elevation and greater electromyographic activity during low-load tasks in comparison with asymptomatic subjects [10,11]). In addition, a significant occurrence of myofascial trigger points (MTrPs) in the levator scapulae muscle is noted in patients suffering from chronic non-traumatic cervical syndrome, cervical radiculopathy, whiplash, and widespread pain syndromes (such as fibromyalgia) [12,13,14,15], producing sensory, motor, and autonomic symptoms [16]. In fact, the prevalence of levator scapulae MTrPs in patients with whiplash injury (76.6%) did not differ significantly from that in patients with fibromyalgia (85.7%) and chronic cervical syndrome (64.7%). It did differ significantly from that in patients with depression (26.7%) and asymptomatic controls (20.8 to 33.6%) [13,14]. The differentiation in levator scapulae MTrPs prevalence between the right and left sides may also indicate potential asymmetries in muscle use or stress, which could be relevant for diagnosis and treatment strategies. This MTrP prevalence rate highlights the importance of the levator scapulae muscle in the context of chronic neck pain and suggests that therapeutic interventions targeting MTrPs in this muscle may be beneficial for a significant subset of patients with chronic musculoskeletal pain.

Ultrasound imaging (US) is a fast, cost-effective, and portable tool used for diagnostics (i.e., assessing the muscle size, shape, quality, and function in other neck muscles [17,18,19]) and therapeutic (i.e., needle guidance for invasive procedures and as a biofeedback tool for facilitating the learning and execution of motor control exercises [20,21]) purposes. In addition, advances in US methods for assessing tissues’ elasticity (i.e., shear-wave elastography, SWE) allow clinicians and researchers to acquire quantitative and objective stiffness data with absolute values (in contrast with strain technology, which is limited to relative information) [22]. Previous studies have used SWE to assess both general muscle stiffness and specific locations within the muscles (including MTrPs) [23]. Although SWE has been demonstrated to be reliable, valid, and objective [22], up to date, the evidence assessing its diagnostic accuracy for assessing the levator scapulae muscle is limited.

Hence, this study aimed to develop a reproducible US protocol for identifying the levator scapulae at a specific location and quantifying its elasticity properties to determine its inter-examiner reproducibility in healthy individuals and patients with mechanical chronic neck pain. As a secondary objective, this research aimed to analyze SWE differences between sides (right and left), genders (males and females), and groups (asymptomatic individuals and patients with bilateral chronic pain). Building upon the literature described in the theoretical framework of our study, we hypothesize that (1) such a protocol would demonstrate consistent inter-examiner reproducibility in both healthy individuals and patients with mechanical chronic neck pain and (2) significant differences in SWE measurements may be observed under these varying conditions.

## 2. Materials and Methods

### 2.1. Study Design

Between November 2022 and April 2023, a cross-sectional observational study with a diagnostic accuracy design was conducted at a private University located in Ávila (Spain). To enhance the writing quality, the study adhered to the Reporting Reliability and Agreement Studies (GRRAS) guidelines [24] and the Enhancing the QUAlity and Transparency Of health Research (EQUATOR) guidelines [25]. In addition, the ethical considerations and study protocol were revised, approved, and supervised by the Ethics Committee of Rey Juan Carlos University prior to starting the data collection.

### 2.2. Participants

Informative local announcements were posted around the Faculty of Health Sciences targeting the recruitment of two samples, one consisting of asymptomatic volunteers and one with chronic idiopathic neck pain. The only general inclusion criterion was to be aged between 18 and 65 years. Those subjects reporting bilateral neck pain with a traumatic origin (e.g., whiplash or fractures), taking medication that may affect muscle tone (i.e., muscle relaxants), with previous surgical procedures, any neuropathic condition (i.e., radiculopathy, thoracic outlet syndrome, or myelopathy), or severe degenerative radiologic findings were excluded from the study. The participants who were eligible for the asymptomatic cohort had to report a lack of neck pain symptoms during the previous year, while participants for the cases cohort were required to report a minimum mean pain intensity of 4 points (greater scores of 3.5 points out of 10 are considered as moderate) in the visual analogue scale [26]. All participants pre-selected for participation in the study were required to read and sign an informed written consent before being enrolled in the data collection.

### 2.3. Sample Size Calculation

The minimum sample size for this study was estimated according to the guidelines presented by Walter et al. [27], which are based on intraclass correlation coefficients (ICCs). The sample size calculation conducted for this study was based on two published reliability studies using US and SWE targeting the levator scapulae muscle in subjects with and without neck pain [28,29], considering an ICC = 0.63–0.99 as the reference range. Considering that inter-examiner reliability is generally poorer than intra-examiner reliability [30], ICC > 0.70 (which is considered indicative of good reliability [28]) was considered the minimally acceptable cut-off.

Given that (1) an expected ICC value of 0.9 was hypothesized based on the B-mode reliability results; (2) a power of 80% and a significance level of 5% were established; and (3) 10% losses were assumed due to the longitudinal nature of the study (participants were examined twice with a considerable time difference between trials), the minimum sample size required for this study was determined to be 65 data points.

### 2.4. Examiners

For conducting this study, one examiner with over 10 years of experience in musculoskeletal ultrasound imaging and 10 years of clinical experience focused on musculoskeletal conditions and chronic neck pain and one novel examiner with 1 year of experience participated in this study, acquiring and measuring all the images. This decision was made to assess the impact of examiners’ experience on measurement agreement, as this selection mirrors real-world clinical scenarios where professionals of varying experience levels often collaborate. To enhance the methodological quality of the study, the participation of individuals and the order of the side examined were randomized. Additionally, a schedule with two rotating shifts (9:00 h to 13:00 h and 15:00 to 17:00 h) was implemented to prevent communication and ensure agreed decisions, rotating the shift each day. On the other hand, participants were asked to attend two appointments within the same day (one in the morning shift and one in the afternoon shift, one with each examiner).

All the images acquired were codified for each examiner, following instructions from the main researcher. Subsequently, the same 2 examiners (experienced and novel) performed all measurements, randomizing the image order (participant and side) and blinded to the examiner who acquired the image (even if they finally measured their own images). This decision was made to provide more pragmatic results in the clinical practice and evaluate the real clinical application of this method, where normally, the same examiner measures the acquired image. Therefore, the blinding process considered the examiner (experienced or novel), participant (case or control), and side (left or right) factors for each image.

### 2.5. Ultrasound Imaging Acquisition Protocol

All US images were collected using a Logiq E9 device with a linear transducer (6–15 MHz ML-6-15-D, General Electric Healthcare, Milwaukee, WI, USA). Standard console settings, including a frequency of 12 MHz, gain of 65 dB, and depth of 4.5 cm, were used for all acquisitions.

Participants were positioned in the prone position with a pillow placed under their ankles, ensuring passively a neutral cranio-cervical position. The upper limbs rested at 90° abduction and 90° of elbow flexion. All participants received instructions to relax their muscles during the exam in order to reduce stiffness variability attributable to muscle contraction.

**Figure 1:**
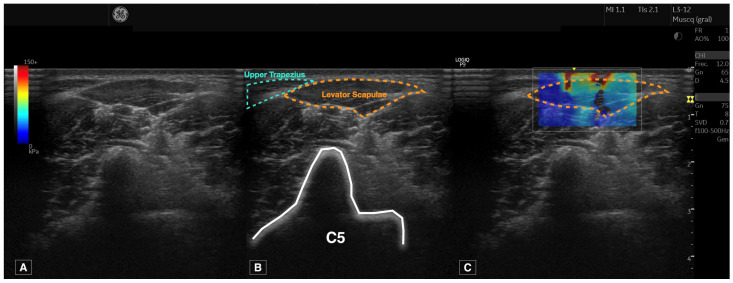
Raw ultrasound imaging acquired at the C5 level (**A**), contouring the targeted structure (levator scapulae) and references used (upper trapezius and transverse process of C5) (**B**) and shear wave elastography imaging (**C**).

The procedure followed to locate the levator scapulae muscle was based on the protocol described by Valera-Calero et al. [28] and performed once by each examiner for each side (n = 2 images per participant by each examiner). After manual palpation of the C2 spinous process, the transducer was placed horizontally to visualize a B-mode short-axis image of C2. A caudal glide was performed until the surface of C5 was located. Finally, a lateral glide was performed until the C5 transverse process posterior tubercle was located. The levator scapulae muscle was identified as the muscle over the posterior tubercle, in the superficial layer, and located laterally to the upper trapezius muscle, as illustrated in Figure 1. Special caution was considered to visualize perpendicularly the muscle in the center of the image, applying the minimum pressure possible.

For side blinding purposes, the upper trapezius muscle was consistently orientated to the left side of the image. The region of interest box width and height were set to cover the levator scapulae muscle completely for later analyses.

### 2.6. Measurement of Muscle Stiffness

All images were analyzed using the Logiq E9 offline software. The process consisted of careful contouring of the levator scapulae perimeter, avoiding the inclusion of bone, nerve roots, or surrounding fascia, as shown in Figure 1. Young’s Modulus and the shear wave speed calculations were automatically provided after contouring the muscle.

### 2.7. Statistical Analyses

Data processing and analysis were performed using the Statistical Package for the Social Sciences (SPSS) version 27 for Mac OS (Armonk, NY, USA), with a two-tailed significance level set at *p* < 0.05. The distribution of continuous variables was verified using histograms and Shapiro–Wilk tests. Variables with *p* < 0.05 were considered non-normally distributed, while those with *p* > 0.05 were deemed normally distributed.

Next, descriptive statistics were used to describe the sociodemographic and US characteristics of the sample, examining gender differences for demographic and US data and between side differences for US data using the Student’s *t*-test with a 95% confidence interval.

For the inter-examiner reliability analyses, six metrics were calculated for Young’s modulus and shear wave speed: (1) the mean average and standard deviation from both examiners, (2) the absolute error between examiners, (3) the intraclass correlation coefficients (ICC_3,2_, 2-way mixed model consistency type), (4) the standard error of measurement (SEM = Standard deviation of the mean average × $\sqrt{1-\mathrm{ICC}}$), and (5) the minimal detectable changes (MDC = SEM × $\sqrt{2}$ × 1.96) [31]. Finally, four Bland–Altman plots (two for cases and two for controls, including Young’s modulus and shear wave speed scores obtained by each examiner, the regression line, and 95% confidence interval) were built to illustrate the agreement between two quantitative measurements.

## 3. Results

Of 71 participants potentially eligible for participation, 4 were excluded due to a previous history of whiplash (n = 1 asymptomatic and n = 3 with neck pain symptoms). Therefore, 67 participants were finally included in the data collection (35 males and 32 females; 33 asymptomatic subjects and 34 participants with neck pain), and a total of 268 images were acquired (n = 134 by each examiner, considering that this count includes one image from both the left and right sides for each participant). As described in Table 1, 52.2% were male, 49.3% were classified as asymptomatic individuals, and 51.7% were patients with chronic neck pain symptoms (VAS = 6.1 ± 1.6). No pain intensity differences between genders (males = 6.1 ± 1.4 and females = 6.1 ± 1.9) were reported (*p* = 0.923). US images were acquired for all participants and sides (n = 228, 114 by each examiner), and none of the images acquired were dropped.

As summarized in Table 1, males were significantly taller (*p* < 0.001) and heavier (*p* = 0.003) but had similar age and body mass index (*p* > 0.05). Individuals with neck pain and asymptomatic subjects were comparable in terms of age, height, weight, and BMI (all, *p* > 0.05). The analysis of the levator scapulae elasticity properties across various groups, including gender (males-females) and cohorts (cases-controls), revealed no significant side-to-side differences (all, *p* > 0.05). This absence of significant asymmetry in SWE measurements between both sides has been summarized in Table 1, which presents the mean average values for both sides to simplify the results and analyses. Furthermore, the study found no significant differences in muscle stiffness between male and female participants (*p* > 0.05), nor between individuals with and without neck pain (*p* > 0.05).

**Figure 2:**
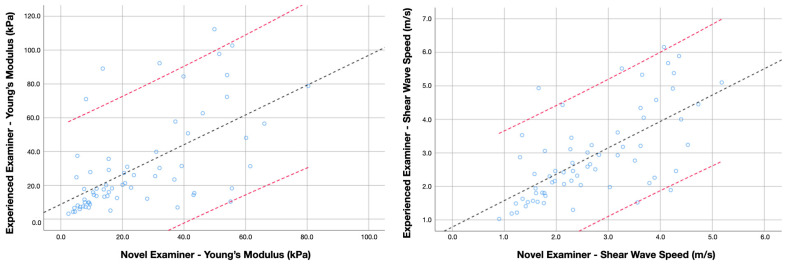
Bland–Altman plot comparing Young’s modulus and shear wave speed scores obtained by the experienced and novel examiner in asymptomatic subjects. Lines represent the regression line (black) and the 95% confidence interval (red).

**Figure 3:**
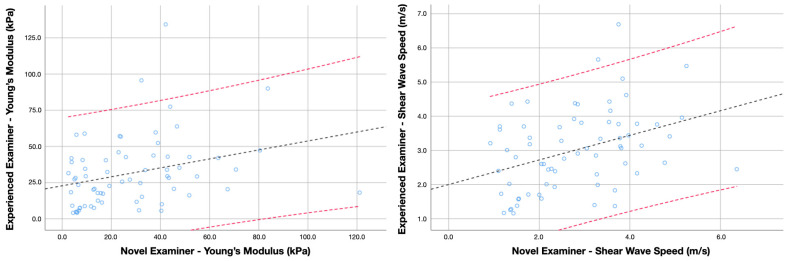
Bland–Altman plot comparing Young’s modulus and shear wave speed scores obtained by the experienced and novel examiner in patients with chronic neck pain. Lines represent the regression line (black) and the 95% confidence interval (red).

Inter-examiner reliability estimates for assessing the levator scapulae muscle stiffness in asymptomatic subjects and patients with neck pain are summarized in Table 2. The analyses revealed that Young’s modulus and shear wave speed obtained by the experienced and the novel examiners did not differ significantly in the asymptomatic cohort (*p* = 0.169 and 0.297, respectively) nor for the sample of patients with neck pain (*p* = 0.374 and 0.297, respectively). However, the absolute error revealed significantly greater disagreement in determining the shear wave speed in the sample of neck pain patients (*p* = 0.045). Accordingly, the ICC scores were moderate-to-good for measuring both metrics in the sample of asymptomatic individuals, while the inter-examiner reliability for the same procedure in patients with chronic neck pain was poor-to-moderate. Additional Bland-Altman plots illustrating the agreement for measuring Young’s Modulus and shear wave speed in healthy individuals and patients with neck pain are available in Figure 2 and Figure 3, respectively.

Finally, the SEM, MDC, and CV are also described in Table 2. In general, the results demonstrated a wide standard deviation, suggesting considerable variability. Regarding the SWE accuracy to determine whether score differences in longitudinal studies are attributable to real changes instead of measurement errors, the analyses revealed greater accuracy for shear wave speed than Young’s modulus.

## 4. Discussion

Although the first hypothesis proposed in this study, expecting acceptable inter-examiner reliability, was built based on promising results in other muscles [32], this hypothesis had to be partially refused after analyzing the results obtained in this study. Even if inter-examiner reliability was good for assessing asymptomatic individuals, the agreement between the two examiners was not acceptable for the case group. Similarly, since the general upper trapezius stiffness was significantly different in asymptomatic subjects and patients suffering from chronic neck pain [23] and based on the high prevalence of MTrP in chronic musculoskeletal pain conditions [12,13,14,15], we expected significant elasticity properties differences between both groups. Despite this literature support, the initial hypothesis was totally refused.

Muscle stiffness is considered an important outcome for clinicians who manage patients with musculoskeletal pain since MTrPs are classically defined as hard palpable nodules within a taut band, and this tender location is associated with the patient’s pain [33]. A previous study [23] aimed to assess the ability of SWE to identify and differentiate between active and latent MTrPs in individuals with unilateral chronic neck pain. For this purpose, the authors included patients with active MTrPs, as well as asymptomatic individuals with latent MTrPs (assessing distal control points with no presence of MTrPs). The objectives were twofold: first, to analyze the differences in pain pressure thresholds (PPTs) and SWE parameters between active MTrPs, latent MTrPs, and control points; and second, to investigate the association of SWE features with clinical severity indicators like pain extent, pain intensity, and neck disability.

The findings of this study were significant in several aspects. Firstly, it was observed that while there were notable differences in the PPTs between active MTrPs and control points (indicating increased pain sensitivity in active MTrPs in accordance with previous meta-analyses [34]), there were no stiffness differences detected by SWE between the active and latent MTrPs or control points. This suggests that while active MTrPs are more sensitive to pain, their stiffness, as measured by SWE, is not distinct from latent MTrPs or normal muscle tissue. Additionally, neck pain patients exhibited increased stiffness in control point locations compared to asymptomatic subjects, highlighting a potential general increase in muscle stiffness associated with neck pain. However, the SWE did not show a significant correlation with clinical severity indicators, indicating its limitations in distinguishing between active and latent MTrPs based on stiffness measures [23].

These findings are in accordance with a randomized clinical trial [35] evaluating the effects of real and sham dry needling on muscle stiffness and PPTs at active MTrPs of the upper trapezius muscle in patients with unilateral chronic neck pain. The trial aimed to compare the immediate effects of a single session of real versus sham dry needling on these parameters. The results revealed that patients receiving real dry needling experienced greater increases in control point PPTs immediately after the intervention compared with the sham group, but no significant differences were found for the MTrPs. Both interventions led to improvements in the PPTs at both MTrP and control point locations, indicating an immediate analgesic effect. However, no significant changes in SWE metrics (shear wave speed and Young’s modulus) were observed at either MTrP or control locations in either group. This suggests that while dry needling can provide immediate pain relief, it does not induce detectable changes in muscle stiffness at the puncture site or distal locations.

Although this is not a study targeting the levator scapulae muscle US assessment [28,29,36,37,38,39], this is the first study (up to the authors’ knowledge) investigating the SWE inter-examiner reliability (based on a previous reliable B-mode procedure for identifying the levator scapulae muscle [27]) for assessing the levator scapulae elasticity properties. In general, we found that the levator scapulae stiffness was comparable, analyzing one cohort of asymptomatic subjects and one of individuals with chronic neck pain with similar sociodemographic characteristics. In addition, no side-to-side stiffness differences were found in any of the groups or genders. Regarding the reliability findings, the procedure was acceptably reliable if asymptomatic subjects were involved (ICC > 0.70), while the same procedure in patients with neck pain symptoms did not reach enough agreement to support its use (ICC < 0.55).

These reliability differences with the reference study [28] could be explained by the study design (this is an inter-examiner reliability study involving a novel examiner while the other study was an intra-examiner reliability study involving a single experienced examiner), sample size (while a sample size calculation was conducted in this study, the reference study had a limited sample size of 25 subjects with no statistical power estimation), and characteristics (since BMI and age were suggested to play a relevant role in US reproducibility [40]), device brands involved, and imaging modes used (panoramic B-mode US vs. SWE).

Since the results obtained in this study suggest that the levator scapulae muscle stiffness has considerable variability, score differences between asymptomatic subjects and patients with neck pain or changes induced by specific interventions should be interpreted cautiously. For instance, Kuo et al. [36] attempted to analyze the stiffness of a series of neck muscles in 3 patients with chronic neck pain and 17 asymptomatic subjects. In contrast with other neck muscles (i.e., sternocleidomastoid, anterior scalene, and upper trapezius), the levator scapulae stiffness showed a non-normal distribution. In addition, other limitations disclosed by the authors, such as the limited sample size and the lack of reliability analyses (as the procedure followed was different from the one described in this report), should be acknowledged.

Considering the poor reliability estimates found in this study following this procedure, a preferable option might be the SWE exploration in a long-axis view, as reported by Yanase et al. [37]. This procedure showed ICC values ranging from 0.63 to 0.96 and CV values ranging from 10.9% to 17.3%. However, these reliability estimates reflect the test-retest reliability of a single examiner with unknown experience in a sample of 15 asymptomatic young men. Therefore, further research should explore this procedure in larger sample sizes, including clinical populations, in order to corroborate its recommendation. An alternative hypothesis explaining the poor inter-examiner reliability found in this study is the role of the examiner experience in acquiring the images and reliably contouring the levator scapulae muscle. A previous study using B-mode ultrasound concluded that a single experienced examiner could reliably identify and measure the cross-sectional area of this muscle (ICC = 0.990) [28]. However, this hypothesis should be confirmed in future studies including at least two experienced examiners and clinical populations (since that ICC score was obtained in asymptomatic subjects [28]) to explain if this lack of agreement can be attributable to a lack of experience or histological-related difficulties (blurred interfaces due to greater intramuscular connective tissues associated with chronic pain syndromes [41]) for contouring the muscle.

In this regard, Tas et al. [38] compared the elasticity of the levator scapulae muscle between patients with and without chronic neck pain (35 participants in each group) using the longitudinal exploration by placing the transducer at the plumpest section of the muscle between the superior angle of the scapula and the midpoint between the transverse processes of C1 and C4. In accordance with our results, they found this muscle to be the most dispersed in terms of shear wave speed. However, they found significant differences between the cases and controls (even if their sample reported lower pain intensity values at baseline in comparison with our sample), reporting a shear wave speed of 4.0 ± 0.8 and 3.7 ± 0.6 m/s, respectively. Despite these significant differences between groups, their correlation analyses revealed no significant associations between the levator scapulae stiffness and pain intensity or neck disability.

In addition, one key finding of this study is the heightened sensitivity of SWS to detect real changes in muscle elasticity in comparison with Young’s modulus. The hypothesis supporting this finding is the direct nature of SWS measurements [42]. While Young’s modulus is an effective parameter for assessing tissue stiffness, it is a derived measure calculated from SWS and density. This calculation can introduce variability, especially in heterogeneous tissues such as striated muscle, potentially reducing sensitivity to subtle changes.

After discussing the results found in this study with the literature exposed, new questions should be approached in future studies. First, a diagnostic accuracy comparison between the short and long axis is needed in asymptomatic and clinical populations, identifying those disagreement contributors (i.e., patient characteristics, examiner expertise, and device algorithms). In addition, since SWE is still considered the most objective method for assessing muscle stiffness (as the hardness of subcutaneous adipose tissues and superficial muscle layers may affect the palpation and stiffness estimation) [40], future studies may assess the clinical relevance of muscle stiffness by analyzing its correlation with other clinical severity indicators (e.g., central sensitization inventory, temporal summation, conditioned pain modulation, pressure pain thresholds, and pain extent).

### Limitations

One such limitation is the number of examiners and device brands involved in this study. Therefore, we cannot be certain whether the poor reliability estimates are related to the examiners’ experience or the devices’ algorithms for calculating the elasticity metrics. Secondly, we only assessed one cervical level in a single short-axis view. Additional research comparing multiple cervical levels in the short and long-axis views is needed. Additionally, we only conducted a single measurement per examiner; therefore, future studies could explore whether an increased number of trials and calculating a mean average of these measurements would enhance the inter-examiner reliability. Finally, since the aim of this research was to analyze the procedure’s reliability, there were no MTrP prevalence assessments or classifications. Further research for analyzing differences between cases and controls should consider following manual and SWE-based protocols for verifying whether MTrPs may influence elasticity differences between groups and their association with clinical severity indicators.

## 5. Conclusions

This study followed a described procedure for locating the levator scapulae muscle and analyzing its elasticity properties assessed with SWE in cohorts with and without chronic neck pain. The obtained results showed that men and women with similar age and BMI are characterized by comparable levator scapulae elasticity. Similarly, individuals with and without neck pain (and similar sociodemographic characteristics) showed no significant levator scapulae elasticity differences. Inter-examiner reliability was moderate to good for assessing asymptomatic subjects while assessing patients with neck pain, which was unacceptably reliable. The variability in results between asymptomatic and symptomatic individuals emphasizes the need for cautious interpretation of stiffness measurements and suggests further research to improve the technique’s reliability, especially in diverse clinical populations. The study also identifies the greater sensitivity of shear wave speed over Young’s modulus in detecting changes in muscle elasticity, pointing towards a preference for shear wave speed measurements in future clinical assessments and research.

## Figures and Tables

**Table 1:** Participants’ sociodemographic and US characteristics.

	Sociodemographic Characteristics				Levator Scapulae SWE ^a^	
	Age (y)	Height (m)	Weight (kg)	BMI (kg/m^2^)	Young’s Modulus (kPa)	Shear Wave Speed (m/s)
Gender						
Males (n = 35)	22.2 ± 4.7	1.77 ± 0.07	74.8 ± 13.3	23.9 ± 3.6	29.6 ± 19.4	2.92 ± 0.99
Females (n = 32)	20.6 ± 2.6	1.64 ± 0.06	65.1 ± 12.5	24.3 ± 4.7	28.4 ± 21.2	2.78 ± 1.10
*Difference*	1.6 (−0.31; 3.5) *p* = 0.101	0.13 (0.09; 0.16) *p* < 0.001	9.7 (3.3; 16.1) *p* = 0.003	0.4 (−1.6; 2.4) *p* = 0.707	1.1 (−5.9; 8.1) *p* = 0.747	0.14 (−0.21; 0.50) *p* = 0.436
Cases and controls						
Asymptomatic subjects (n = 33)	21.4 ± 4.8	1.72 ± 0.08	72.4 ± 14.4	24.3 ± 4.0	27.8 ± 21.4	2.78 ± 1.10
Patients with neck pain (n = 34)	21.5 ± 2.8	1.69 ± 0.10	68.3 ± 13.1	23.8 ± 4.3	29.6 ± 19.1	2.90 ± 1.00
*Difference*	0.0 (−1.9; 2.0) *p* = 0.973	0.03 (−0.01; 0.08) *p* = 0.133	4.1 (−2.7; 10.8) *p* = 0.231	0.4 (−1.6; 2.5) *p* = 0.667	1.8 (−5.1; 8.7) *p* = 0.605	0.11 (−0.24; 0.47) *p* = 0.525

^a^ Reported values are calculated as the mean average of the scores obtained by both examiners.

**Table 2:** Inter-examiner reliability for the stiffness assessment of the levator scapulae muscle in asymptomatic individuals.

Variables	Asymptomatic Individuals (n = 33)		Patients with Neck Pain (n = 34)	
	Young’s Modulus (kPa)	Shear Wave Speed (m/s)	Young’s Modulus (kPa)	Shear Wave Speed (m/s)
Mean (n = 268 images)	27.8 ± 21.4	2.78 ± 1.10	29.6 ± 19.1	2.90 ± 1.00
Experienced examiner (n = 134 images)	30.7 ± 28.4	2.89 ± 1.32	31.4 ± 24.0	3.00 ± 1.17
Novel examiner (n = 134 images)	24.9 ± 19.3	2.67 ± 1.09	27.8 ± 23.3	2.79 ± 1.23
Absolute difference	14.7 ± 8.4	0.72 ± 0.36	19.5 ± 10.2	1.01 ± 0.50
ICC_3,2_ (95% CI)	0.714 (0.533; 0.825)	0.779 (0.639; 0.865)	0.461 (0.127; 0.668)	0.546 (0.265; 0.720)
SEM	11.4	0.51	14.0	0.67
MDC_95_	31.7	1.43	38.9	1.86
CV (%)	52.8	25.9	65.9	34.8

SEM and MDC_95_ are expressed in the units described for each metric.

## Data Availability

All data derived from this study are presented in the text.
